# Author Correction: The kinase TNIK is an essential activator of Wnt target genes

**DOI:** 10.1038/s44318-025-00393-5

**Published:** 2025-07-09

**Authors:** Tokameh Mahmoudi, Vivian S W Li, Ser Sue Ng, Nadia Taouatas, Robert G J Vries, Shabaz Mohammed, Albert J Heck, Hans Clevers

**Affiliations:** 1 Hubrecht Institute – KNAW and University Medical Centre Utrecht, Uppsalalaan 8, 3584CT Utrecht, The Netherlands; 2 Biomolecular Mass Spectrometry and Proteomics Group, Bijvoet Center for Biomolecular Research and Utrecht Institute for Pharmaceutical Sciences, Utrecht University, Padualaan 8, 3584 CH Utrecht, The Netherlands; 3 Netherlands Proteomics Centre, Utrecht, The Netherlands; 4 Centre for Biomedical Genetics, Utrecht, The Netherlands

## Abstract

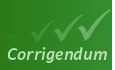

**Correction to:** *The EMBO Journal* (2009) 28:3329–3340. 10.1038/emboj.2009.285 | Published online 8 October 2009

After being informed of a potential microduplication in Figure 1D, the journal contacted the authors for clarification. The authors provided the original source data, which confirms that no duplication occurred in the original dataset.

For transparency, the original source data is published with this author correction.

Author Statement:

In the original publication, an error was identified in Figure 1 *(IP TCF4, WB β-catenin in the villus compartment)*. A duplication occurred, resulting in a small, duplicated segment at the end of the panel.

The original source data has been located and is provided with this author correction. The experiment was performed in February 2009, as indicated on the films. Two different exposures of the same panel are included—one unannotated and one with annotations for clarification.

The original data confirms that a duplication was present only in the published figure; however, no data was obscured by this duplication. The data clearly shows the absence of interaction between TCF4 and beta-catenin in the villus compartment (confirming our mass spectrometry data and expected given the absence of Wnt signalling in the villus compartment).

This correction does not alter the conclusions of the study.

## Supplementary information

Original data corresponding to beta catenin panel Figure 1 with annotation.

Scan of films only corresponding to beta catenin panel Figure 1.

