# Supplementary figures and images for: Author Correction: The kinase TNIK is an essential activator of Wnt target genes
Source: EMBO J. 2025 Jul 9;44(15):4407. doi: 10.1038/s44318-025-00393-5 (PMC12316840; doi:10.1038/s44318-025-00393-5)

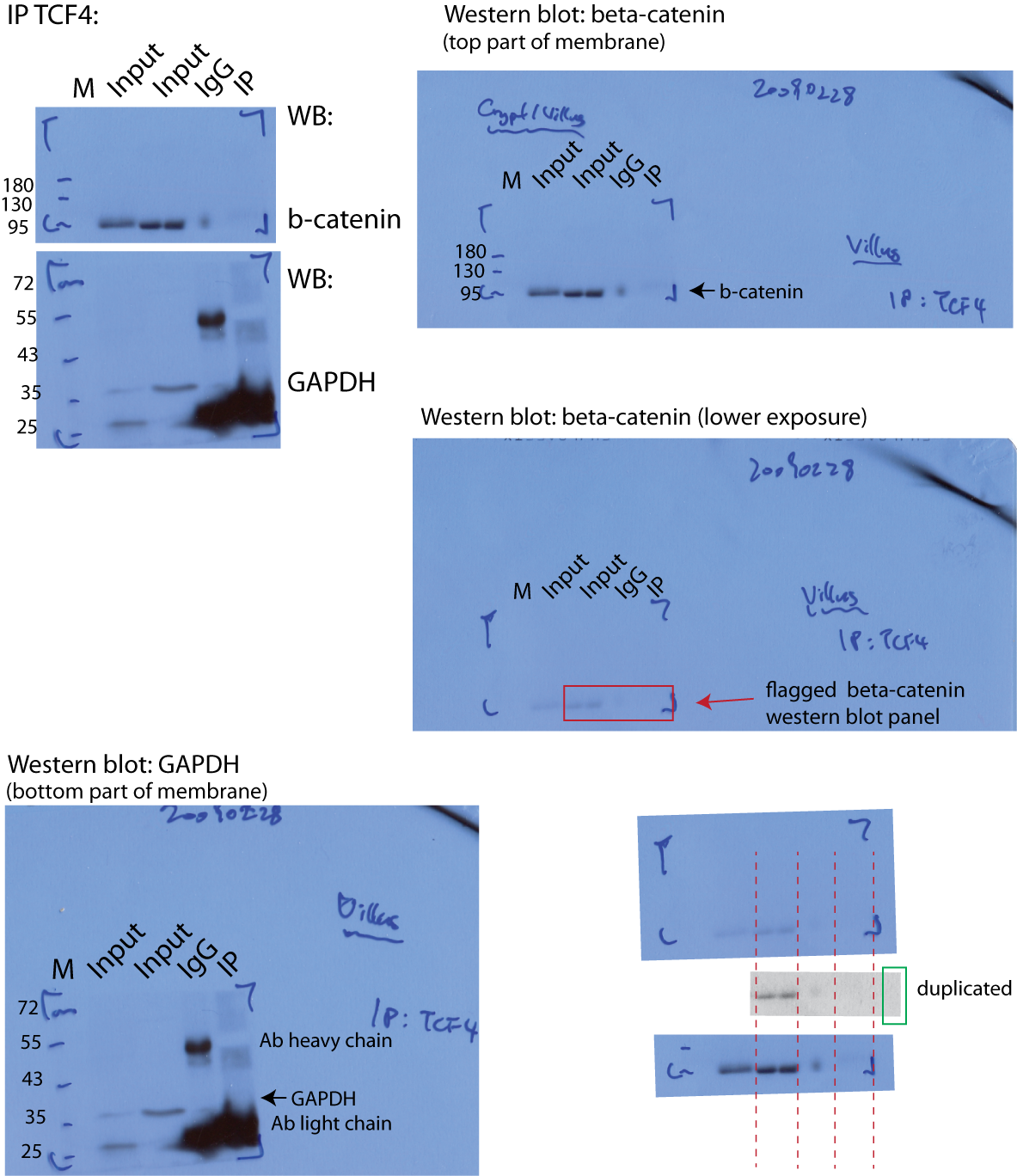

Supplement: Supplementary file 1 — Original data corresponding to beta catenin panel Figure 1 with annotation. [file 44318_2025_393_MOESM1_ESM.tif]

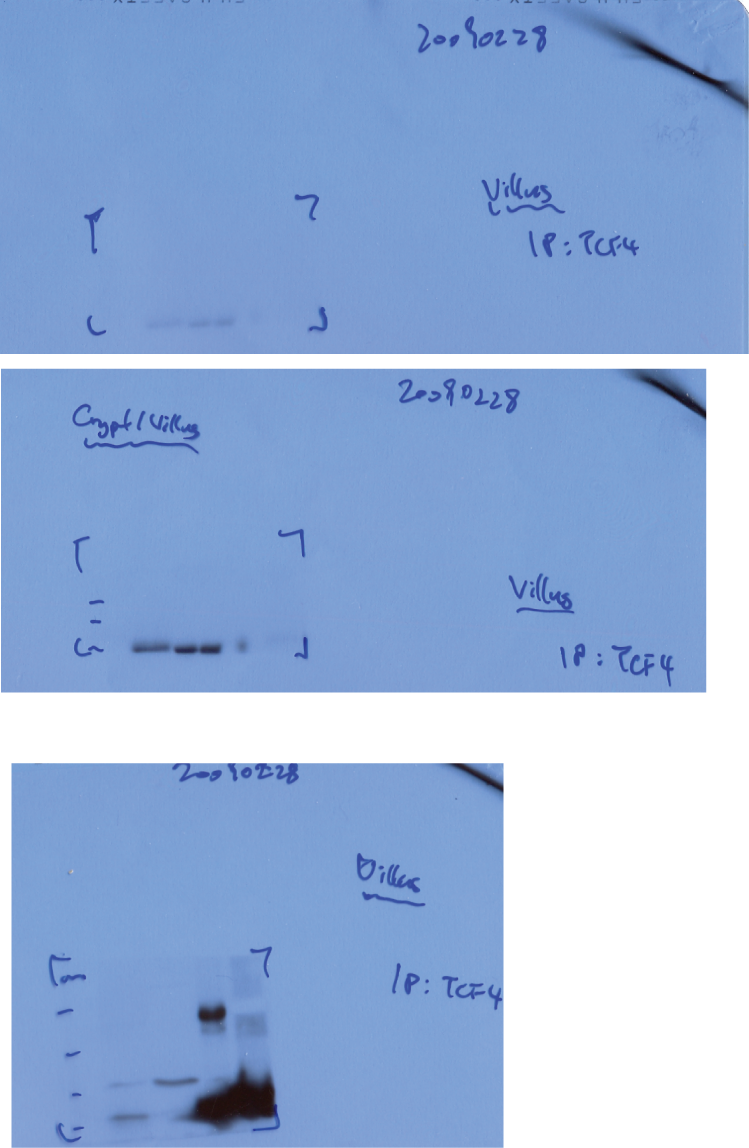

Supplement: Supplementary file 2 — Scan of films only corresponding to beta catenin panel Figure 1. [file 44318_2025_393_MOESM2_ESM.tif]
